# Response competition between neurons and antineurons in the mushroom body

**DOI:** 10.1016/j.cub.2021.09.008

**Published:** 2021-11-22

**Authors:** Eleftheria Vrontou, Lukas N. Groschner, Susanne Szydlowski, Ruth Brain, Alina Krebbers, Gero Miesenböck

**Affiliations:** 1 Centre for Neural Circuits and Behaviour, University of Oxford, Tinsley Building, Mansfield Road, Oxford OX1 3SR, UK

## Abstract

The mushroom bodies of *Drosophila* contain circuitry compatible with race models of perceptual choice. When flies discriminate odor intensity differences, opponent pools of αβ core Kenyon cells (on and off αβ_c_ KCs) accumulate evidence for increases or decreases in odor concentration. These sensory neurons and “antineurons” connect to a layer of mushroom body output neurons (MBONs) which bias behavioral intent in opposite ways. All-to-all connectivity between the competing integrators and their MBON partners allows for correct and erroneous decisions; dopaminergic reinforcement sets choice probabilities via reciprocal changes to the efficacies of on and off KC synapses; and pooled inhibition between αβ_c_ KCs can establish equivalence with the drift-diffusion formalism known to describe behavioral performance. The response competition network gives tangible form to many features envisioned in theoretical models of mammalian decision making, but it differs from these models in one respect: the principal variables—the fill levels of the integrators and the strength of inhibition between them—are represented by graded potentials rather than spikes. In pursuit of similar computational goals, a small brain may thus prioritize the large information capacity of analog signals over the robustness and temporal processing span of pulsatile codes.

## Highlights

• Mushroom body output neurons respond with excitation to odor on- and offset
• On and off responses reflect the convergence of oppositely tuned Kenyon cells (KCs)
• Opponent KCs compete by eliciting inhibitory feedback from a common interneuron pool
• KCs and interneurons communicate through graded potentials rather than spikes

## Teaser

Vrontou et al. demonstrate the convergence of oppositely tuned Kenyon cell (KC) populations onto the same mushroom body output neurons (MBONs). The rival KCs vie for control of MBON activity via independently plastic synapses and inhibitory feedback from a common interneuron pool, as proposed in response competition models of perceptual choice.

## Introduction

Two-alternative forced-choice tasks, in which a subject must commit to one of two alternatives, sometimes under time pressure and nearly always with uncertain information, are a commonly studied laboratory simplification of real-world decision making. The neural processes that culminate in a binary choice have been compared to the deliberations of a jury before a verdict:^1^ neurons, like jurors, gather evidence from witnesses over the course of a trial and then reconcile their divergent views in a majority vote.

The problem of how neural circuits implement this form of trial by jury has been approached in a range of species, from primates and rodents to fish and flies. A pioneering and influential body of work is built on a two-alternative forced-choice task in which monkeys distinguish directions of motion in a noisy random dot display.^2^ Recordings of correlated neuronal activity suggest that motion-sensitive neurons in the middle temporal visual area (MT or V5) provide momentary evidence^2^^,^^3^ that is temporally integrated in lateral intraparietal cortex (LIP) before passing an unspecified thresholding mechanism.^4^^,^^5^ Although the precise role attributed to LIP is a matter of debate,^6^ the principle that ephemeral sensory signals flow into integrators whose fill levels rise to a response threshold appears general; similar arrangements have been inferred to support visual motion discrimination in zebrafish^7^^,^^8^ and odor intensity discrimination in the fly.^9^^,^^10^

In *Drosophila*, a rate-limiting integration step takes place in a particular group of third-order olfactory neurons.^9^^,^^10^ When flies decide on the direction of an odor concentration change, the membrane potentials of Kenyon cells (KCs) in the αβ core (αβ_c_) division of the mushroom bodies drift noisily toward action potential threshold,^10^ just as accumulating evidence would drift toward a response bound.^1^^,^^11^, ^12^, ^13^, ^14^ Consistent with the proposed correspondence of membrane voltage and integrated sensory information, and of action potential and decision thresholds, neurometric functions based on the average timing of the first odor-evoked spikes in the αβ_c_ KC population can account for the speed and accuracy of the decision-making animal;^10^ psychophysical estimates of noise in the decision process match the measured membrane potential noise of αβ_c_ KCs;^10^ and genetically targeted manipulations that alter the latencies of αβ_c_ KC spikes have the expected impact on reaction times.^9^^,^^10^

Two functionally separate groups of αβ_c_ KCs, termed up and down or on and off cells, respond to increases or decreases in odor concentration^10^^,^^15^ and can therefore represent the strength of evidence for either of the two alternatives in the choice.^10^ This explicit representation of support for each of the competing hypotheses (as opposed to an aggregate representation of the extent to which one hypothesis is favored over the other) suggests that a decision involves a race between two integrators^12^^,^^16^^,^^17^—one built from neurons that accumulate evidence for an increase in odor concentration and another composed of “antineurons”^2^ that do the opposite. Changes in odorant receptor occupancy at the periphery alter the baseline activity of olfactory receptor neurons and the second-order projection neurons (PNs) with which they form receptor-specific glomerular channels. Large odor concentration changes in a channel’s preferred direction drive high-frequency transmission from PNs to αβ_c_ KCs that promotes steep depolarizations to spike threshold and fast, accurate decisions, whereas small concentration changes in the preferred direction, or any change in the null direction, cause only a trickle of synaptic release; shallow, undulating membrane potential rises; and long spike delays that lead to slow, error-prone choices.^10^^,^^14^

In this study, we examine whether the circuitry downstream of αβ_c_ KCs is compatible with a model of two competing integrators. We test three predictions of such a model. First, to adjudicate the rival hypotheses advocated by on and off αβ_c_ KCs, mushroom body output neurons (MBONs)^18^^,^^19^ sampling the cores of the αβ lobes must listen to both. We therefore expect that each core-innervating MBON is excited by increases as well as decreases in odor concentration. Second, as an animal learns the rules of the two-alternative forced-choice task—that an increase in odor intensity predicts imminent electric shock, whereas a decrease signals protection^9^—the influence of αβ_c_ KCs championing the correct choice should be enhanced while that of proponents of the incorrect choice should be diminished. In other words, we expect antagonistic changes in the strengths of connections of on and off αβ_c_ KCs with the same action selection neurons if evidence for the competing alternatives is accumulated separately. Third, race models become equivalent to a drift-diffusion process—the formalism shown accurately to describe the psychophysics of the decision-making animal^9^^,^^10^—only if they include an element of mutual or pooled inhibition^12^^,^^20^^,^^21^ to establish response competition between the integrators.^22^ Inhibition is needed to ensure that the integrators are anti-correlated so that evidence for one choice simultaneously counts as evidence against the other. We therefore predict the existence of inhibitory interactions between αβ_c_ KCs.

## Results

### Core-innervating MBONs respond to odor on- and offset

Seven classes of KC (of which αβ_c_ KCs are one) elaborate parallel, tightly packed axon bundles that fill the cross-sections of the mushroom body lobes.^18^^,^^19^ As they run the lengths of each lobe, KC axons cross five or six compartments defined by the non-overlapping dendritic fields of MBONs.^19^ Each compartment functions as a semi-autonomous memory unit that records the predictive value of the animal’s olfactory experience for a set of motivated actions. Information is stored when local dopaminergic reinforcement adjusts the connection strengths of KCs with the compartment’s inherently valued MBON.^14^^,^^19^^,^^23^, ^24^, ^25^, ^26^, ^27^, ^28^ As in an electronic memory, matched pairs of data input and output lines—dopaminergic neurons (DANs) and MBONs, respectively—supply each data storage location.^19^^,^^23^

The system is arranged so that MBONs driving approach read KC activity in the vertical (α and α’) lobes, peduncle, and heel (i.e., the γ1 compartment), while MBONs driving aversion sample the horizontal (β, β’, and γ) lobes.^25^ This arrangement is mirrored in the segregation of positively and negatively reinforcing DANs: the axonal projections of negatively reinforcing neurons in the paired posterior lateral cluster 1 (PPL1) target the vertical lobes and heel,^23^^,^^29^ while those of rewarding neurons in the paired anterior medial cluster (PAM) are confined to the horizontal lobes.^30^^,^^31^ Because coincident dopamine release onto active KC-to-MBON synapses causes synaptic depression,^24^^,^^26^ PPL1 reinforcement diminishes an odor’s appeal by attenuating how strongly its KC representation activates attractive MBONs, while PAM reinforcement does the opposite by weakening KC coupling to aversive outputs.

**Figure 1:**
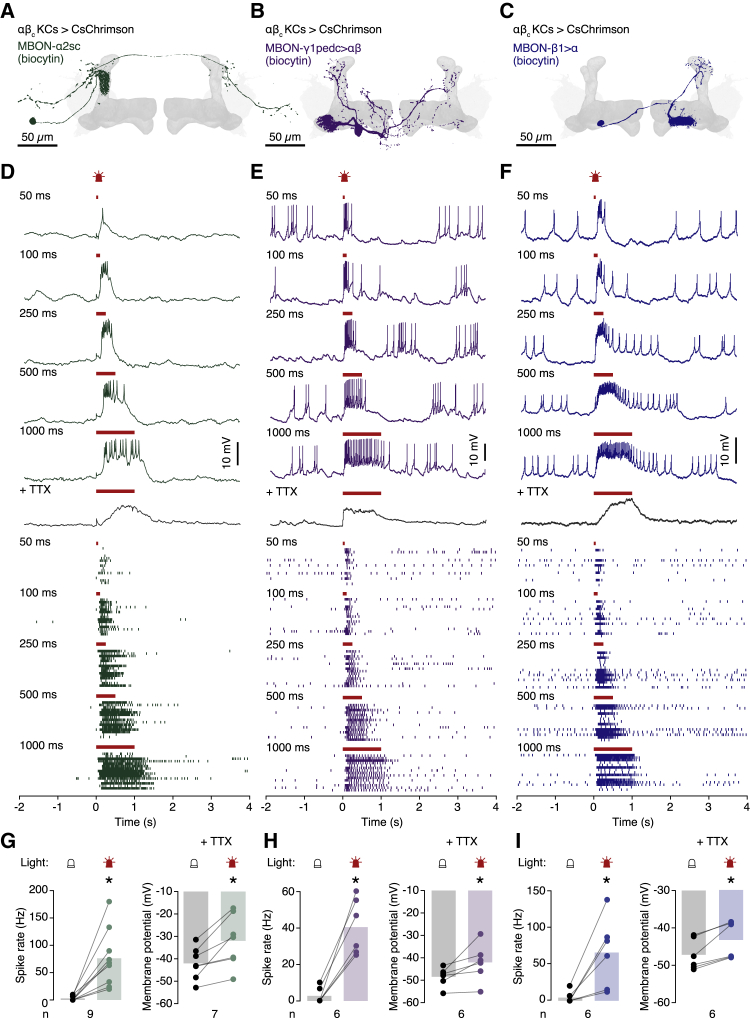
Three types of core-innervating MBON (A–C) Biocytin fills of MBON-α2sc (A), MBON-γ1pedc>αβ (B), and MBON-β1>α (C). (D–F) Example voltage traces (top) and spike rasters (bottom) of MBON-α2sc (D), MBON-γ1pedc>αβ (E), and MBON-β1>α (F) during optogenetic activation of αβ_c_ KCs for the indicated durations. (G–I) Average spike rates (left) and membrane potentials (right) before and during optogenetic activation of αβ_c_ KCs, in the absence (left) or presence of TTX (right). Illumination increased the spike rates and membrane potentials of MBON-α2sc (G; p = 0.0312 and p = 0.0084; Wilcoxon test and paired t test, respectively), MBON-γ1pedc>αβ (H; p = 0.0312 and p = 0.0469; Wilcoxon test and paired t test, respectively), and MBON-β1>α (I; p = 0.0312 and p = 0.0312, respectively; Wilcoxon tests). ^∗^p < 0.05. Data are means ± SEM; n, number of cells. See also Figure S1.

Anatomical analyses^19^^,^^32^ suggest that αβ_c_ KCs are a major, but not necessarily the only, source of presynaptic input to three MBON types: MBON-α2sc in the α lobe (Figure 1A); MBON-γ1pedc>αβ in the peduncle (Figure 1B); and MBON-β1>α in the β lobe (Figure 1C). We verified the existence of functional connections in targeted whole-cell recordings from these MBONs *in vivo*, using selective *GAL4* drivers to label individual neurons (Figures 1 and S1). Different output neurons showed characteristic rates and patterns of baseline activity, ranging from quiescence or sparse firing (MBON-α2sc; Figure 1D) to persistent irregular spiking or bursting (MBON-γ1pedc>αβ and MBON-β1>α; Figures 1E and 1F) or alternating UP and DOWN states (MBON-γ4>γ1γ2, which lacks demonstrable core innervation^19^^,^^32^ and served as our specificity control; Figures S1A and S1B). These different MBON personalities have approximate counterparts in the corresponding DANs (see below).

Optogenetic activation^33^ of αβ_c_ KCs expressing CsChrimson caused depolarizations of up to 20 mV that elevated the firing rates of all three core-innervating MBONs (Figures 1D–1I), but not of MBON-γ4>γ1γ2 (Figures S1B and S1C). Blocking voltage-gated sodium channels with tetrodotoxin (TTX) eliminated action potentials but preserved the voltage deflection on which these action potentials normally ride (Figures 1D–1I), while adding the nicotinic acetylcholine receptor antagonist mecamylamine^34^ on top of TTX leveled also the subthreshold depolarization and thereby ruled out leaky expression of the optogenetic actuator in the MBON itself (Figures S1D–S1F).

**Figure 2:**
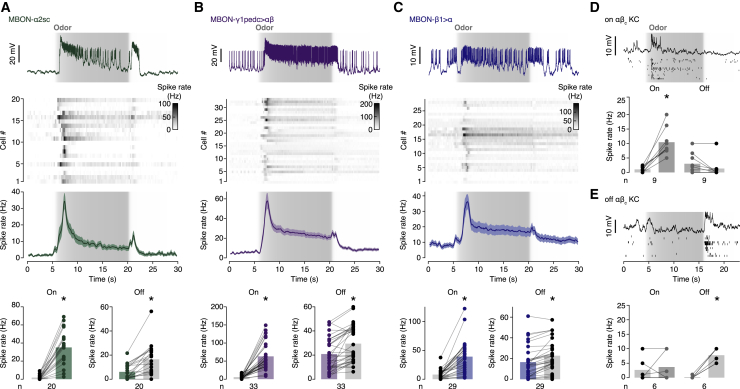
Responses of core-innervating MBONs to odor on- and offset (A–C) Responses of MBON-α2sc (A), MBON-γ1pedc>αβ (B), and MBON-β1>α (C) during 15-s odor pulses (gray shading indicates measured concentration time courses). Top to bottom: example voltage traces, heatmaps of the spike rates of individual cells, spike rate averages (±SEM), and spike rate comparisons before and after odor intensity changes. Odor on- and offset increased the spike rates of MBON-α2sc (A; p < 0.0001 and p < 0.0001, respectively; Wilcoxon tests), MBON-γ1pedc>αβ (B; p < 0. 0001 and p < 0.0001, respectively; Wilcoxon tests), and MBON-β1>α (C; p < 0.0001 and p < 0.0001; Wilcoxon test and paired t test, respectively). (D and E) Responses of on (D) and off αβ_c_ KCs (E) during 10-s odor pulses (gray shading indicates measured concentration time courses). Top to bottom: example voltage traces, spike rasters of individual cells, and spike rate changes caused by odor on- and offset. Odor onset, but not odor offset, increased the spike rates of on αβ_c_ KCs (D; p = 0.0039 and p = 0.1250, respectively; Wilcoxon tests), while odor offset, but not odor onset, increased the spike rates of off αβ_c_ KCs (E; p = 0.0312 and p = 0.7500, respectively; Wilcoxon tests). ^∗^p < 0.05. Data are means ± SEM. See also Figure S2.

Long odor pulses elicited voltage and spiking responses in core-innervating MBONs whose sharp onset resembled that during optogenetic core KC activation (Figures 2A–2C). A few seconds into the pulse, after the initial depolarization to peak, the membrane potentials and spike rates declined and stabilized at slightly (MBON-α2sc) or moderately elevated plateaux (MBON-β1>α and MBON-γ1pedc>αβ) before rising again to a second peak at the end of the pulse (Figures 2A–2C). We attribute the peaks at odor on- and offset to transmission dominated by on and off αβ_c_ KCs (Figures 2D and 2E) whose distinct contributions became visible when a long odor pulse separated them widely in time.^15^ Under more realistic conditions, such as short odor exposures resembling concentration fluctuations within an odor plume, the on and off responses bled into each other (Figures S2A and S2B).

Although the numerical excess of on over off αβ_c_ KCs is modest at best,^10^^,^^15^ the off responses of all three MBON types were uniformly smaller than their on responses (Figures 2A–2C). The relative strengths of these responses could simply be handed down to αβ_c_ KCs from their presynaptic partners, or the KC group that becomes active first—here, on αβ_c_ KCs at odor onset—could partially suppress later responders through response competition, as envisioned in our third prediction. Noting this subtlety (but putting it to one side for the moment), we conclude that Figure 2 confirms our first prediction: each core-innervating MBON responds to increases as well as decreases in odor concentration.

### Spike latencies of MBONs versus αβ_c_ KCs

**Figure 3:**
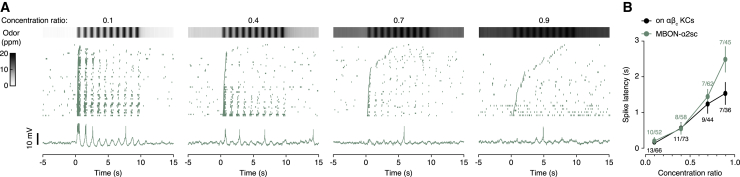
Spike latencies of MBON-α2sc versus αβ_c_ KCs (A) Top to bottom: MCH concentrations, spike rasters, and example voltage traces of MBON-α2sc during ten odor intensity cycles between a variable base (2–18 ppm) and a constant peak (20 ppm) MCH concentration. Measured MCH concentration time courses at the different base-to-peak ratios (0.1–0.9, left to right) are displayed on top. The spike rasters are sorted, in ascending order from the bottom, by the latency of the first spike. (B) Spike latencies of MBON-α2sc and on αβ_c_ KCs as functions of MCH concentration ratio. Data are means ± SEM; sample sizes (cells/trials) are indicated near data points. The spike latencies of αβ_c_ KCs are replotted from Groschner et al.^10^

If αβc KCs perform a rate-limiting integration step in the decision process,^10^ they must be rate limiting also for the odor responses of their postsynaptic partners. To establish this point, we concentrated on MBON-α2sc, whose sparse basal activity (Figure 1D) allowed precise measurements of spike latencies. When flies were exposed to a stream of 4-methyl-cyclohexanol (MCH) switching repeatedly between a base concentration that varied between trials (2–18 ppm) and a peak concentration that remained constant (20 ppm), the latency of the first MBON-α2sc spike increased monotonically with diminishing intensity contrast (Figure 3A). The growing MBON spike delays matched those of αβ_c_ KCs under the same conditions^10^ and paralleled increases in decision time when flies discriminate ever smaller intensity differences.^9^ Averaged across trials, the action potentials of MBON-α2sc closely tracked or slightly trailed—but never led—those of αβ_c_ KCs (Figure 3B), which therefore represent the temporal bottleneck in the process.

An extensive analysis of odor tuning in the KC ensemble showed that any given KC will respond to the onset of some odors and the offset of others (Jan Kropf, Clifford B. Talbot, and G.M., unpublished data). Put differently, the same neuron will be classified as an on or an off cell (but rarely as both)^15^ in different olfactory contexts. Because the KCs responsible for the on and off responses are functionally interchangeable (for example, by choosing different odors), a spike latency match similar to the one we have demonstrated for MCH at odor onset (Figure 3B) is expected to hold true also at odor offset.

### Reciprocal plasticity of on and off responses

Flies learn to perform odor intensity discrimination by analyzing temporal relations between events. If a neutral odor reliably precedes an unpleasant experience, such as an electric shock, the odor itself acquires negative valence: its presentation generates the anticipation of danger and its fading the anticipation of relief, while the converse is true if the sequence of events is reversed.^35^ The physical expression of these changed expectations is altered synaptic weights between KCs and MBONs.^24^^,^^26^, ^27^, ^28^ A second prediction of our model is, therefore, that positive or negative reinforcement of an odor will change the coupling strengths of its on and off αβ_c_ KCs with the same MBONs in opposite directions.

**Figure 4:**
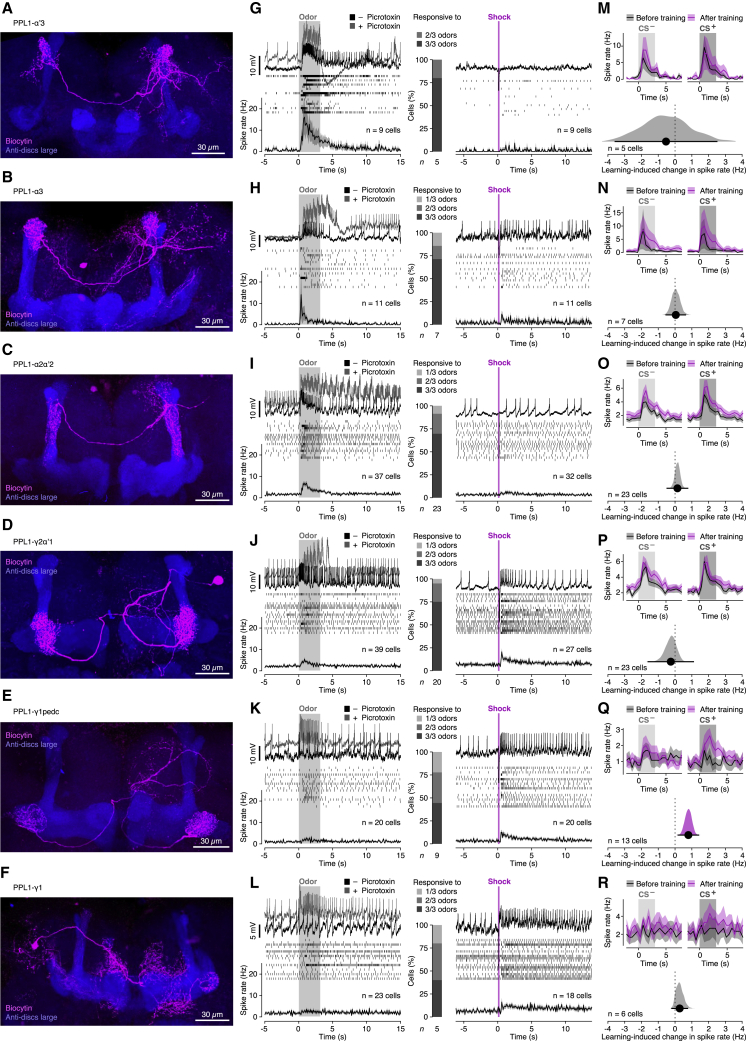
Functional classification of PPL1 neurons (A–F) Biocytin fills of mushroom-body-innervating PPL1 neurons (magenta). Synaptic structures of the mushroom body lobes were counterstained with an antibody against discs large (blue). (G–L) Top to bottom: example voltage traces, spike rasters, and spike rate averages (±SEM) of different types of PPL1 neuron during 3-s exposures to isopentyl acetate (left, gray) or 10-ms exposures to electric shock (right, magenta). Stacked column graphs in the center show response percentages to the odors isopentyl acetate, MCH, and OCT. The left-hand panels include example voltage traces after the addition of picrotoxin (gray). (M–R) Top: spike rate averages (±SEM) of different types of PPL1 neuron during exposures to MCH (CS^−^, left) or OCT (CS^+^, right), before (black) and after (magenta) electric shock reinforcement. Bottom: effect size plots of training-induced spike rate changes in different types of PPL1 neuron: (CS^+^_after_ − CS^+^_before_) − (CS^−^_after_ − CS^−^_before_). Shaded areas with solid bars at their bases represent bootstrapped sample distributions and 95% confidence intervals, respectively. Magenta color indicates a statistically significant training effect only for PPL1-γ1pedc (Q; p = 0.0022; one-sample t test). ^∗^p < 0.05. Data are means ± SEM. See also Figure S3.

Dopaminergic neurons encode the error signals that guide learning when reality and expectation collide.^23^, ^36^, ^37^ Because flies in our two-alternative forced-choice task are trained by unexpected electric shock,^9^ members of the 12-strong PPL1 cluster of negatively reinforcing DANs are the relevant carriers of these signals.^23^ As a prelude to exploring the types of plasticity these neurons instruct, we characterized their activity in whole-cell recordings. On the basis of their axonal projection patterns (Figures 4A–4F), we could distinguish 10 morphological PPL1 types, six of which targeted the mushroom bodies:^19^^,^^38^ the tips of the α’ (PPL1-α’3; Figure 4A) and α lobes (PPL1-α3; Figure 4B); the vertical stalk (PPL1-α’2α2; Figure 4C); the junction between the vertical and horizontal lobes (PPL1-γ2α’1; Figure 4D); the α, β, and γ portions of both heels (PPL1-γ1pedc; Figure 4E); and the γ portion of the contralateral heel (PPL1-γ1; Figure 4F).

The six mushroom-body-innervating PPL1 neurons fell into two broad classes based on their differential sensitivity to punitive cues (Figures 4G–4L). Applications of electric shock caused prominent firing rate increases in one group (PPL1-γ2α’1, PPL1-γ1pedc, and PPL1-γ1; Figures 4J–4L) but had muted effects on the other (PPL1-α’3, PPL1-α3, and PPL1-α’2α2; Figures 4G–4I). Conversely, presentations of odors caused the largest firing rate modulations in cells with the faintest shock responses (Figures 4G–4I). Where odor responses were detected, they were broadly tuned: at least two of the three odors tested elicited spiking in the majority of responsive neurons (Figures 4G–4I). A tentative interpretation of these observations, which mesh with earlier imaging analyses,^38^^,^^39^ is that the PPL1 cluster contains two anatomically and functionally separable dopaminergic systems: a group of sporadically firing cells (PPL1-α’3, PPL1-α3, and PPL1-α’2α2), which report sudden changes in sensory input (novelty or salience),^39^ and a group of more persistently active neurons (PPL1-γ1pedc, PPL1-γ1, and PPL1-γ2α’1), which offer a running commentary on the animal’s hedonic state (valence).

Closer scrutiny, however, revealed that the separation between odor- and pain-responsive cells was not absolute. Cases in point are PPL1-γ1pedc and PPL1-γ1, which often showed, in addition to prominent spike rate increases after electric shock, muffled odor responses that could be boosted substantially by blocking GABA_A_ receptors with picrotoxin (Figures 4K, 4L, and S3A). GABA exerted at least some of its inhibitory effect directly on these DANs, as spatially restricted RNA-mediated interference (RNAi) with the expression of the GABA_A_ receptor Rdl also amplified their odor responses (Figure S3B). All PPL1 neurons thus receive broadly tuned excitatory olfactory input, but balanced inhibition normally suppresses the responses of valence-encoding cells to behaviorally neutral odors.

Formal accounts of learning posit that neurons carrying reinforcement signals become responsive to neutral stimuli after these stimuli acquire motivational significance (for example, as predictors of reward or pain).^36^ The presence of broadly tuned odor responses that are normally concealed by inhibition suggests a simple mechanism for transferring activity from a primary reinforcer to a conditioned stimulus: odor-specific changes in the balance of excitation and inhibition could unmask the suppressed response to the conditioned odor. Because the pain-responsive DANs are latent odor generalists (Figures 4J–4L), such a mechanism could rapidly associate any odor with a motivational dopamine signal.

**Figure 5:**
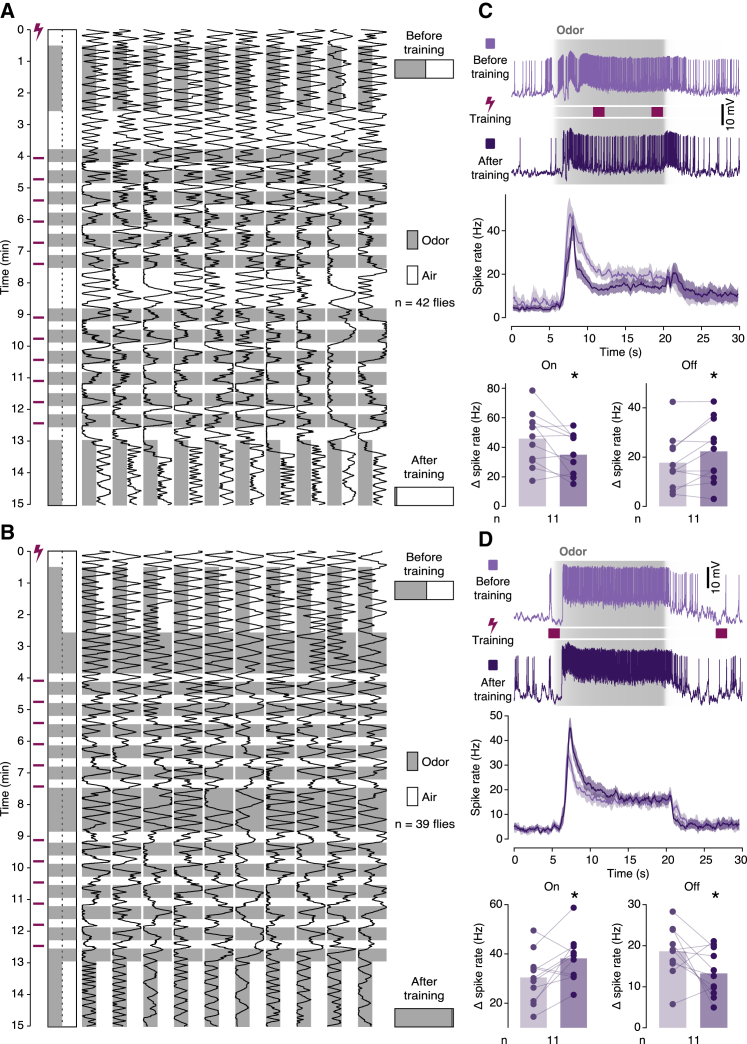
Reciprocal plasticity of on and off responses (A and B) Behavioral plasticity induced by electric shock reinforcement after odor onset (A) or odor offset (B). Example movement traces of 10 flies in individual chambers. The flies were allowed to choose between MCH (gray) and air during test periods before and after the electric shock reinforcement of MCH (A) or air (B), as diagrammed on the left. Bar graphs on the right indicate the fractions of choices in favor of MCH (gray) and air (white) before and after training. (C) Synaptic plasticity induced by electric shock reinforcement after odor onset. Responses of MBON-γ1pedc>αβ to 15-s odor pulses (gray shading indicates measured concentration time courses), before and after electric shock reinforcement (light and dark colors) at times indicated by magenta bars. Top to bottom: example voltage traces, spike rate averages (±SEM), and spike rate changes caused by odor on- and offset. The on and off responses were depressed and potentiated, respectively (p = 0.0262 and p = 0.0325; paired t tests), after training. (D) Synaptic plasticity induced by electric shock reinforcement after odor offset. Responses of MBON-γ1pedc>αβ to 15-s odor pulses (gray shading indicates measured concentration time courses), before and after electric shock reinforcement (light and dark colors) at times indicated by magenta bars. Top to bottom: example voltage traces, spike rate averages (±SEM), and spike rate changes caused by odor on- and offset. The on and off responses were potentiated and depressed, respectively (p = 0.0050 and p = 0.0471; paired t tests), after training. ^∗^p < 0.05. Data are means ± SEM. See also Figures S4 and S5.

To examine whether learning would indeed expose or augment a cryptic dopamine response to the conditioned odor,^40^ we recorded from PPL1 neurons during conditioning trials that paired two blocks of six 3-s presentations of the odor 3-octanol (OCT) with applications of electric shock. Presentations of OCT with shock reinforcement were interlaced with presentations of a second odor, MCH, without reinforcement. Five minutes after the last training cycle, we challenged the animals with the two odors in random sequence. Of the six mushroom-body-innervating PPL1 neurons (Figures 4M–4R), only PPL1-γ1pedc showed enhanced responses to the conditioned odor after training (Figure 4Q). MBON-γ1pedc>αβ, the output neuron twinned with PPL1-γ1pedc, therefore took center stage in our search for reciprocal changes in its coupling strengths to on and off αβ_c_ KCs that are expected to underlie short-term changes in behavior (Figures 5A and 5B). We obtained current-clamp recordings from MBON-γ1pedc>αβ and exposed flies to a total of six 15-s odor pulses that each overlapped with two 1.5-s epochs of electric shock. This training protocol altered the on and off responses of MBON-γ1pedc>αβ antagonistically, in keeping with our second prediction: the on response was depressed while the off response was potentiated (Figures 5C and S4A).

### Plasticity rules

The differential plasticity of on and off responses is a likely consequence of the order in which on and off αβ_c_ KCs become active relative to PPL1-γ1pedc: the activation of on αβ_c_ KCs precedes the shock-induced dopamine release, whereas the activation of off αβ_c_ KC trails it (Figures 5A and 5C). KC-to-MBON synapses are sensitive to the timing of dopaminergic reinforcement and undergo depression or potentiation after forward or backward pairing.^28^ Applying these rules to the dual representation of odors by on and off KCs could explain the antagonistic changes in the MBON’s on and off responses: because each odor is represented twice (once by on and once by off αβ_c_ KCs; Figure 2), it is also conditioned twice—forward at onset and backward at offset (Figures 5A and 5C). Inverting these timing relationships (by delivering electric shock during the air interval between odor pulses; Figures 5B and 5D) turned learned aversion into attraction (compare Figures 5A and 5B) and produced mirror-symmetric changes in the on and off responses of MBON-γ1pedc>αβ (compare Figures 5C and 5D).

**Figure 6:**
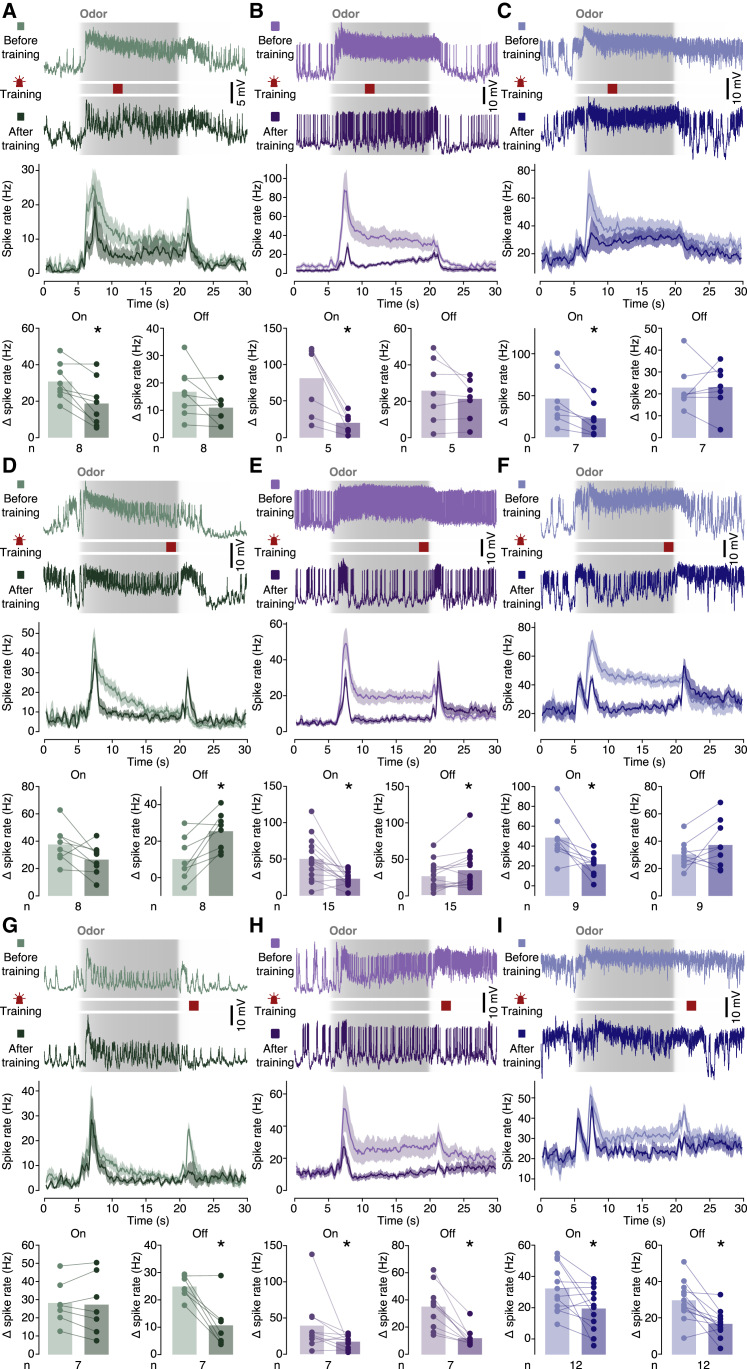
Plasticity rules Responses of MBON-α2sc (left column), MBON-γ1pedc>αβ (center column), or MBON-β1>α (right column) to 15-s odor pulses (gray shading indicates measured concentration time courses), before and after the optogenetic activation of the affine PPL1 or PAM neurons (light and dark colors) at times indicated by crimson bars. Top to bottom: example voltage traces, spike rate averages (±SEM), and spike rate changes caused by odor on- and offset. (A–C) Dopaminergic reinforcement after odor onset depressed all on responses (A, p = 0.0277; B, p = 0.0156; C, p = 0.0172; paired t [A and C] or Wilcoxon tests) but left the off responses unchanged (A, p = 0.0764; B, p = 0.2537; C, p = 0.9596; paired t tests). (D–F) Dopaminergic reinforcement before odor offset potentiated most off responses (D, p = 0.0056; E, p = 0.0256; F, p = 0.2100; paired t [D and F] or Wilcoxon tests) and depressed most on responses (D, p = 0.0709; E, p = 0.0023; F, p = 0.0064; paired t tests). (G–I) Dopaminergic reinforcement after odor offset depressed all off responses (G, p = 0.0312; H, p = 0.0020; I, p = 0.0019; paired t [I] or Wilcoxon tests) as well as some on responses (G, p = 0.6690; H, p = 0.0273; I, p = 0.0248; paired t [G and I] or Wilcoxon tests). ^∗^p < 0.05. Data are means ± SEM.

To examine whether similar plasticity rules held for all core-innervating MBONs, we replaced the electric shock reinforcer with direct optogenetic activation of the affine DANs: of PPL1-γ1pedc for MBON-γ1pedc>αβ, of PPL1-α’2α2 for MBON-α2sc, and of PAM neurons for MBON-β1>α. Nesting a block of optogenetic reinforcement within the 15-s odor pulse preserved the sequential activation of on KCs, DANs, and off KCs and recapitulated the pattern of plastic changes seen during standard electric shock training (Figure 5C); it produced depressed on and potentiated off responses in MBON-α2sc (Figure S5A) and MBON-γ1pedc>αβ (Figures S4B and S5B). Confining reinforcement to the first few seconds of the odor pulse selectively depressed the on response but left the off response unchanged (Figures 6A–6C), whereas restricting reinforcement to the period immediately before odor offset potentiated most off responses while continuing to depress some on responses (Figures 6D–6F). The efferent synapses of KCs therefore remained plastic long after the peak in odor-evoked activity, as is evident also behaviorally: flies correctly credited pain to predictive odor intensity changes experienced some 18 s earlier (Figures 5A and 5B). Consistent with a slowly decaying eligibility trace in αβ_c_ KC terminals, DAN activation after odor offset depressed not only the off but sometimes also the still pliant on response (Figures 6G–6I).

### Inhibitory competition between αβ_c_ KCs

The division between on and off αβ_c_ KCs does not respect fixed anatomical or gene expression boundaries but instead changes dynamically with sensory input (Jan Kropf, Clifford B. Talbot, and G.M., unpublished data). This fluid, odor-specific membership of individual neurons in the two functional KC classes, and the consequent lack of genetic markers for these classes, made an experimental test of our third prediction—that on and off αβ_c_ KCs inhibit one another—difficult.

**Figure 7:**
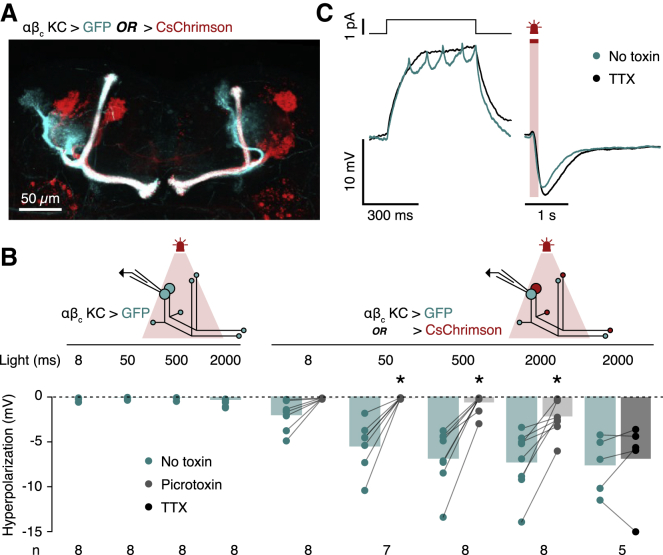
Inhibition between αβ_c_ KCs (A) Mutually exclusive FLP-mediated expression of CD8::GFP (cyan) or CsChrimson::tdTomato (crimson) in αβ_c_ KCs. (B) Peak hyperpolarizations of CD8::GFP-positive αβ_c_ KCs during light pulses of the indicated durations, in the absence (left) or presence (right) of FLP-mediated recombination to generate a CsChrimson-expressing αβ_c_ KC population, before (green columns) or after the addition of picrotoxin (light gray columns) or TTX (dark gray column). Two-way repeated-measures ANOVA detected significant effects of light exposure and picrotoxin on the amplitude of hyperpolarization (p = 0.0034 and p < 0.0001, respectively), while TTX had no effect (p = 0.6250; Wilcoxon test). ^∗^p < 0.05 in post hoc comparisons. Data are means ± SEM. (C) Example voltage responses of a CD8::GFP-positive αβ_c_ KC during a 500-ms depolarizing current step (left) and a 200-ms light pulse (right), before and after the addition of TTX (green and black traces, respectively). See also Figure S6.

To circumvent this difficulty, we generated a GAL4-responsive FLP-out construct^41^ for the mutually exclusive expression of CsChrimson or GFP in random subsets of αβ_c_ KCs. We then used these non-overlapping populations of differentially responsive neurons as proxies for on and off KCs (in the original context, on and off KCs are differentially responsive to odor concentration changes; here, the two complementary KC sets are differentially responsive to light). The CsChrimson-expressing population was also tagged with tdTomato, which permitted us to visualize the two KC sets and verify the absence of overlap between them (Figure 7A). Recombination, mediated by low-level uninduced expression of an *hsFLP* transgene,^42^ typically took place at the neuroblast stage, with all descendants of each of the four lineages that give rise to the mushroom body^43^ belonging to one or the other αβ_c_ KC set (Figure 7A). As predicted, current-clamp recordings from GFP-positive (and, therefore, CsChrimson-negative) αβ_c_ KCs showed deep, photon dose-dependent hyperpolarizations of up to 15 mV below the membrane potential baseline when the CsChrimson-positive complement of αβ_c_ KCs was stimulated by light (Figures 7B and S6). Picrotoxin blocked the inhibitory response, identifying it as GABAergic (Figure 7B). Because KCs themselves are cholinergic and excitatory,^34^ within-core inhibition must involve an intermediary that signals through GABA, is activated by KCs, and provides feedback inhibition to KCs. The anterior paired lateral neuron (APL), a GABAergic neuron present in a single copy per hemisphere, fits this bill precisely.^18^^,^^32^^,^^44^^,^^45^ APL forms reciprocal synapses with KCs throughout the mushroom body^18^^,^^32^ and delivers local, KC division- or compartment-restricted inhibition.^44^, ^45^, ^46^

The subthreshold nature of the integration process,^10^ however, poses a conundrum: if on and off αβ_c_ KCs accumulate sensory evidence by integrating synaptic potentials to spike threshold,^10^ how can APL exert inhibitory feedback when neither competitor emits an action potential before the race is decided? A likely answer lies in the peculiar structure and biophysics of the feedback loop. αβ_c_ KCs and APL are densely interconnected not only in the mushroom body lobes, which contain the axons of KCs, but also in the calyx, where KC dendrites reside.^18^ Here, both neuronal partners form numerous presynaptic active zones,^32^^,^^47^ indicative of bidirectional communication between them: each αβ_c_ KC excites APL through an average of 13.4 synapses—that is, roughly one contact per 20 μm of dendrite—and is inhibited by APL via 10.0 reciprocal synapses.^46^ Like its locust analog, the giant GABAergic neuron (GGN), APL is a non-spiking cell whose rate of GABA release tracks local voltage changes in a graded fashion;^48^ in GGN and other non-spiking interneurons, depolarizations of 2–5 mV suffice to trigger secretion.^48^^,^^49^

But how is transmission at the dendritic release sites^32^^,^^47^ of αβ_c_ KCs controlled? Membrane potential measurements suggest that these synapses also function in analog, graded-potential mode, whereas transmission at axonal KC-to-MBON terminals is digital and spike dependent. Action potentials backpropagating from the axon initial segment of αβ_c_ KCs arrived at the somatic recording site with severely attenuated amplitudes of 1.95 ± 0.09 mV (mean ± SEM; n = 34 cells), barely larger than those of single excitatory postsynaptic potentials (EPSPs) advancing through the dendritic tree (mean ± SEM = 1.25 ± 0.05 mV; n = 36 cells). In the dendrites themselves (which are inaccessible to direct measurements), the relative magnitude of the potentials may flip, with the now unattenuated EPSPs exceeding the size of backpropagating spikes. EPSPs, not action potentials, would then be the dominant form of depolarization in KC dendrites and, as such, the principal drivers of transmitter release. Indeed, the light-induced hyperpolarizations of CsChrimson-negative αβ_c_ KCs persisted unabated when action potentials were blocked with TTX (Figures 7B and 7C). All circuit elements are therefore in place for pooled inhibition^12^^,^^20^, ^21^, ^22^ between on and off αβ_c_ KCs, with APL representing the interneuron “pool” fed by the competing integrators.

## Discussion

The idea that decisions are based on the accumulated spikes of oppositely tuned sensors was born in early attempts to unite psychophysical and neurophysiological measurements under the umbrella of signal detection theory.^2^^,^^3^ The recorded spike count distributions of direction-selective units in the monkey’s area MT to motion in the preferred or null directions were taken to represent the responses of two neurons—the recorded neuron and its imagined antineuron conjugate—to movement in the neuron’s preferred direction. The likely direction of motion can then be inferred as the probability that a draw from the neuron’s response distribution yields a larger spike count than a draw from the antineuron’s.^2^^,^^3^ At minimal motion strengths, when the two distributions are congruent, these odds are even and choices are random, but as the neuron responds ever more vigorously to increasingly coherent motion while the antineuron’s response stays flat, the distributions unmix and the probability of a correct choice rises toward one. Comparing the spike counts of two sensors rather than thresholding the output of one removes shared sources of variation and with them the need of adjusting the discrimination threshold to achieve the best separation of the changing response distributions: a neuron-antineuron pair always returns a quantity proportional to likelihood ratio, the optimal hypothesis test.^1^^,^^11^ Although opponent sensory channels in one or another guise feature prominently in many decision-making models,^1^, ^2^, ^3^^,^^11^^,^^12^^,^^14^^,^^20^, ^21^, ^22^^,^^50^ their involvement in the brain is unproven: neurons and antineurons owe their status to each other, as inputs to comparator circuits, but these circuits remain uncharacterized.

Our study draws back the curtain on one such circuit in the fly. Changes in odor intensity are registered by pools of on and off αβ_c_ KCs, which represent the strengths of the accumulated evidence for an increase or decrease in odor concentration.^10^ These pools of sensory neurons and antineurons couple to a second layer of neurons and antineurons, the core-innervating MBONs (Figures 1 and 2), which bias behavioral intent in opposite ways.^25^ Members of both neuronal pools in the sensory layer connect to both types of MBON in the action selection layer via plastic synapses (Figures 2 and 6). With two sets of neurons and antineurons and all-to-all feedforward connectivity between them (Figure 2), the comparator circuit allows for approach or avoidance following judgments of upward or downward changes in odor intensity—that is, it comprises neural pathways representing the possible contingencies seen behaviorally (Figure 5). The perceptual decision is won—correctly or incorrectly—by the αβ_c_ KC pool that reaches spike threshold first (Figure 3), and it is expressed in the behavior instructed by that pool’s favored MBON partners.

Unlike neurons comprising the ON and OFF pathways of motion vision,^51^^,^^52^ on and off αβ_c_ KCs cannot be distinguished and manipulated genetically. We have therefore exploited the sensitivity of KC-to-MBON synapses to the timing of reinforcement^28^ to reveal the convergence of separate on and off channels onto the same MBONs. KC-to-MBON synapses in their ground state exert finely balanced drive on the MBON ensemble, so that votes cast by its members cancel one another as in a hung jury, but experience can shift the synaptic weight distribution and the resulting pattern of MBON activation away from net zero.^25^^,^^27^ We have documented such shifts for the approach-advocating^25^ MBON-γ1pedc>αβ: pairing odor on- or offset with electric shock weakens transmission from the on αβ_c_ KC pool and strengthens transmission from the off αβ_c_ KC pool (or vice versa), synergistically changing odor preference (Figure 5). The underlying mechanism is a switch from synaptic depression to synaptic potentiation^28^ when the order of odor-evoked KC activation and dopaminergic reinforcement is reversed (Figure 5). This mechanism operates at KC connections with all core-innervating MBONs but is likely engaged at different timescales that may reflect sequential memory phases; to demonstrate the mechanism’s ubiquity, we have artificially collapsed this temporal sequence by photostimulating DANs directly (Figure 6). Within the short time frame of our behavioral experiments (Figure 5), only PPL1-γ1pedc, but not PPL1-α’2α2, shows significant pain responses that modulate its sensitivity to a predictive odor (Figure 4), consistent with the view that PPL1-γ1pedc and its cognate MBON-γ1pedc>αβ represent the core circuit for the storage and expression of short-term aversive memories.^25^^,^^29^

A crucial element of many neural network models of decision making is inhibitory feedback from a common interneuron pool driven by the competing integrators,^12^^,^^20^, ^21^, ^22^ which helps to amplify small differences in conflicting sensory evidence until, eventually, one integrator prevails. The response competition circuit we have delineated contains such an inhibitory element but with the intriguing twist that the key variables are represented by membrane voltages rather than spikes (Figure 7).^10^ Analog processing may be a consequence of numerical constraints: if the mushroom bodies lack the neuron numbers needed to approximate continuous quantities with discrete-time action potentials,^53^ there may be little choice but to swap the advantages regenerative spikes could provide (such as long time windows for adding and retaining sensory evidence)^53^ for the greater information capacity of graded potentials.^54^^,^^55^ Perhaps more *is* different.^56^

## STAR★Methods

### Key resources table

**Table:** 

REAGENT or RESOURCE	SOURCE	IDENTIFIER
**Antibodies**		

Mouse monoclonal 4F3 anti-discs large	Developmental Studies Hybridoma Bank, University of Iowa	RRID: AB_528203
AlexaFluor568-conjugated goat anti-mouse IgG	Invitrogen (Thermo Fisher)	A-11031 RRID: AB_144696

**Chemicals, peptides, and recombinant proteins**		

Biocytin	Sigma-Aldrich	B4261
AlexaFluor633-conjugated streptavidin	Invitrogen (Thermo Fisher)	S21375
Paraformaldehyde	Electron Microscopy Sciences	15713
Phosphate buffered saline tablets	Oxoid	BR0014G
Vectashield antifade mounting medium	Vector Laboratories	H-1000
4-methyl-cyclohexanol (MCH)	Sigma-Aldrich	153095
3-octanol (OCT)	Sigma-Aldrich	218405
Isopentyl acetate	Sigma-Aldrich	306967
Ethyl acetate	Sigma-Aldrich	270989
Eicosane	Sigma-Aldrich	219274
Thermoplastic wax (52°C melting point)	Agar Scientific	AGG3881
TES	Sigma-Aldrich	T5691
NaCl	Sigma-Aldrich	S7653
KCl	Sigma-Aldrich	P9333
NaHCO_3_	Sigma-Aldrich	S6297
NaH_2_PO_4_	Sigma-Aldrich	S8282
CaCl_2_	Sigma-Aldrich	21115
MgCl_2_	Sigma-Aldrich	M1028
Trehalose	Sigma-Aldrich	T9531
Glucose	Sigma-Aldrich	G7528
Sucrose	Sigma-Aldrich	S0389
HEPES	Sigma-Aldrich	H4034
MgATP	Sigma-Aldrich	A9187
Na_3_GTP	Sigma-Aldrich	G6129
EGTA	Sigma-Aldrich	E4378
all-*trans* retinal	Sigma-Aldrich	R2500
Dimethyl sulfoxide	Sigma-Aldrich	D2650
Tetrodotoxin citrate	Tocris	1069
Picrotoxin	Tocris	1128
Mecamylamine hydrochloride	Sigma-Aldrich	M9020

**Experimental models: organisms/strains**		

*Drosophila,* Canton-S	Bloomington Drosophila Stock Center	64349
*Drosophila, hsFLP*	Bloomington Drosophila Stock Center^42^	26902
*Drosophila, NP6024-GAL4*	Kyoto Stock Center^18^	105080
*Drosophila, NP7175-GAL4*	Kyoto Stock Center^18^	114120
*Drosophila, R58F02-GAL4*	Bloomington Drosophila Stock Center^57^	39186
*Drosophila, MB112C-GAL4*	Bloomington Drosophila Stock Center^19^	68263
*Drosophila, MB80C-GAL4*	Bloomington Drosophila Stock Center^19^	68285
*Drosophila, MB433B-GAL4*	Bloomington Drosophila Stock Center^19^	68324
*Drosophila, MB320C-GAL4*	Bloomington Drosophila Stock Center^26^	68253
*Drosophila, MB099C-GAL4*	Bloomington Drosophila Stock Center^19^^,^^26^	68290
*Drosophila, R12G04-LexA*	Bloomington Drosophila Stock Center^26^	52448
*Drosophila, R58E02-LexA*	Bloomington Drosophila Stock Center^31^	52740
*Drosophila, R34B02-LexA*	Bloomington Drosophila Stock Center^26^	53631
*Drosophila, UAS-CD8::GFP*	Bloomington Drosophila Stock Center^58^	32186
*Drosophila*, *UAS-Rdl.RNAi.8-10*	Bloomington Drosophila Stock Center^59^	89903
*Drosophila, UAS-Dcr-2*	Bloomington Drosophila Stock Center^60^	24650
*Drosophila, 10xUAS-IVS-Syn21-Chrimson::tdT-3.1 (su(Hw)attP1) “UAS-CsChrimson::tdTomato”*	Gift from D. Anderson^61^	N/A
*Drosophila, 20xUAS-IVS-CsChrimson.mVenus (attp40) “UAS-CsChrimson::mVenus”*	Bloomington Drosophila Stock Center^62^	55135
*Drosophila, lexAop-rCD2::GFP*	Bloomington Drosophila Stock Center^63^	66544
*Drosophila, 13xLexAop2-IVS-CsChrimson.mVenus (attp2) “lexAop-CsChrimson”*	Bloomington Drosophila Stock Center^62^	55139
*Drosophila, 13xLexAop-IVS-GFP-p10 (su(Hw)attP5) “lexAop-GFP”*	Gift from G. Rubin^64^	N/A
*Drosophila, 10x-UAS-FRT > -IVS-mCD8::GFP-STOP-FRT > -IVS-CsChrimson::tdTomato (attp40)*	This study	N/A

**Recombinant DNA**		

Plasmid, pJFRC177-10XUAS-FRT>-dSTOP-FRT>-myr::GFP	Addgene^65^	32149

**Software and algorithms**		

MATLAB	Mathworks	https://www.mathworks.com
LabVIEW	National Instruments	https://www.ni.com
ImageJ	NIH	https://imagej.nih.gov
Signal	Cambridge Electronic Design Ltd.	http://ced.co.uk
pClamp 10	Molecular Devices	https://www.moleculardevices.com
Igor Pro	Wavemetrics	https://www.wavemetrics.com
NeuroMatic	NeuroMatic	http://neuromatic.thinkrandom.com
Python 3.7.1	Python Software Foundation	https://www.python.org
pyABF 2.2.3	Harden Technologies	https://pypi.org/project/pyabf/
dabest 0.3.1		https://pypi.org/project/dabest/
Prism	GraphPad	https://www.graphpad.com

**Other**		

Borosilicate glass capillaries	Sutter Instruments	BF150-86-10

### Resource availability

#### Lead contact

Requests for resources and reagents should be directed to and will be fulfilled by the lead contact, Gero Miesenböck (gero.miesenboeck@cncb.ox.ac.uk).

#### Materials availability

All unique reagents generated in this study are available from the lead contact.

### Experimental model and subject details

Experimental flies were heterozygous for all transgenes. For patch-clamp recordings, the expression of *UAS-CD8::GFP*^58^ was targeted to MBON-α2sc, MBON-γ1pedc>αβ, MBON-β1>α, or MBON-γ4>γ1γ2 using *MB080C-GAL4*, *MB112C-GAL4*, or *MB433B-GAL4*, respectively;^19^ to PPL1 neurons using *TH-GAL4*;^66^ or to αβ_c_ KCs using *NP6024-GAL4, NP7175-GAL4,* or *R58F02-GAL4*.^18^^,^^57^ To deplete the GABA_A_ receptor Rdl from PPL1 neurons, *MB320C-GAL4*^26^ controlled the expression of *UAS-Rdl.RNAi.8-10*^59^ and *UAS-Dcr-2.*^60^

In combined photostimulation and whole-cell recording experiments, the following transgene combinations were used to drive the expression of optogenetic actuator and fluorescent marker:^18^^,^^19^^,^^26^^,^^57^^,^^58^^,^^61^, ^62^, ^63^, ^64^ αβ_c_ KCs (*R58F02-LexA* > *lexAop-CsChrimson*) and MBONs (*MB080C-GAL4*, *MB112C-GAL4*, or *MB433B-GAL4>UAS-CD8::GFP*); PPL1-α’2α2 (*MB099C-GAL4>UAS-CsChrimson::tdTomato*) and MBON-α2sc (*R34B02-LexA>lexAop-GFP*); PPL1-γ1pedc (*MB320C-GAL4>UAS-CsChrimson::mVenus*) and MBON-γ1pedc>αβ (*R12G04-LexA>lexAop-rCD2::GFP*); and PAM-β1 DANs (*R58E02-LexA>lexAop-CsChrimson*) and MBON-β1>α (*MB433B-GAL4>UAS-CD8::GFP*). Not all drivers exhibited absolute specificity for the neurons of interest:^19^^,^^26^^,^^31^ *MB433B-GAL4* expresses in both MBON-β1>α and MBON-γ4>γ1γ2; *MB099C-GAL4* labels PPL1-α’2α2 and PPL1-γ2α’1 and stochastically PPL1-α’3 and/or PPL1-α3; and *MB320C-GAL4* captures PPL1-γ1pedc and with low frequency also PPL1-α’2α2 and/or PPL1-α’3.

To achieve the mutually exclusive expression of CD8::GFP or CsChrimson::tdTomato in αβ_c_ KCs, a *CD8::GFP* coding sequence followed by the SV40 transcriptional terminator was flanked by *FRT* sites and inserted between the *UAS* promoter and the *CsChrimson::tdTomato* coding sequence, using *pJFRC177-10XUAS-FRT>-dSTOP-FRT>-myr::GFP*^65^ as the backbone. The construct was integrated into the *attp40* site and transcribed under the control of *R58F02-GAL4.*^57^ The transcript encoded CD8::GFP by default and CsChrimson::tdTomato after the FLP-mediated excision^41^ of the *FRT>–CD8::GFP–stop–FRT>*cassette. Low levels of basal expression of the *hsFLP* transgene, without additional heat shock, were sufficient to produce recombination events^42^ whose visible signature was a mosaic of green and red fluorescent αβ_c_ KCs (Figure 7A). The presence of both KC populations (or the absence of red fluorescence in flies lacking *hsFLP*) was confirmed by live microscopy before each experiment. Recordings from CsChrimson-positive αβ_c_ KCs showed that the membrane potentials of these neurons were correctly modulated by light (Figure S6).

Fly strains were grown on standard cornmeal agar under a 12 h light:12 h dark cycle at 25°C unless they carried the *hsFLP* transgene; these experimental animals and their controls were raised at 18°C. Flies expressing CsChrimson were transferred to food supplemented with 0.6–2 mM all-*trans* retinal in dimethyl sulfoxide upon eclosion and raised in darkness thereafter.

### Method details

#### Electrophysiology

For whole-cell patch-clamp recordings *in vivo*, male or female flies aged 2–11 days were fixed to a custom mount with eicosane or soft thermoplastic wax (Agar Scientific). Cuticle, adipose tissue, trachea, and glial sheath were surgically removed to expose the brain, which was continuously superfused with extracellular solution (pH 7.3) containing 5 mM TES, 103 mM NaCl, 3 mM KCl, 26 mM NaHCO_3_, 1 mM NaH_2_PO_4_, 1.5 mM CaCl_2_, 4 mM MgCl_2_, 8 mM trehalose, 10 mM glucose, and 7 mM sucrose (275 mOsM, equilibrated with 5% CO_2_ and 95% O_2_). The green-fluorescent somata of target cells (MBONs, PPL1 neurons, αβ_c_ KCs) were visually identified using 40 × , 0.8 NA or 60 × , 1.0 NA water immersion objectives (LUMPLFLN40XW or LUMPLFLN60XW, Olympus) and a combination of epifluorescence and differential interference contrast. Patch pipettes (11–22 MΩ, depending on target cell type) were fabricated from borosilicate glass capillaries with outer and inner diameters of 1.5 and 0.86 mm (Sutter Instruments), using a PC-10 micropipette puller (Narishige) or a DMZ Universal Electrode Puller (Zeitz), and filled with solution (pH 7.3) containing 10 mM HEPES, 140 mM potassium aspartate, 1 mM KCl, 4 mM MgATP, 0.5 mM Na_3_GTP, 1 mM EGTA, and 10 mM biocytin (265 mOsM). Signals were recorded at room temperature (21–23°C) in current-clamp mode with a MultiClamp 700B amplifier (Molecular Devices), lowpass-filtered at 10–20 kHz, and sampled at 20–50 kHz using a Power1401-3A data acquisition interface controlled through Signal (Cambridge Electronic Design Ltd.), an ITC-18 board (InstruTECH) controlled through Igor Pro (WaveMetrics), or a Digidata 1440A digitizer controlled through pCLAMP 10 (Molecular Devices). Data were analyzed with custom procedures, using the NeuroMatic package (http://neuromatic.thinkrandom.com) in Igor Pro (WaveMetrics) and the pyABF module (https://pypi.org/project/pyabf/) in Python 3.7 (Python Software Foundation). Only cells with a measured resting potential below –30 mV and a spiking response to depolarizing current injections were characterized further. Where indicated, mecamylamine (500 μM), picrotoxin (10 μM as standard; up to 250 μM in some cases), or tetrodotoxin (TTX, 1μM) were perfused into the bath to verify cholinergic, GABAergic, or monosynaptic or graded transmission, respectively.

For photostimulation of CsChrimson-expressing cells, a 630-nm LED (Multicomp OSW-4388) controlled by a TTL-triggered dimmable LED driver (Recom RCD-24-0.70/W/X3) was focused on the head of the fly with a mounted 60 mm lens (Thorlabs). The light source delivered 11–80 mW cm^-2^ of optical power.

Custom solenoid valve systems (The Lee Company) under LabVIEW control (National Instruments) directed mass flow-controlled (CMOSens, Sensirion) streams of clean or odor-infused air (containing 1–20 ppm 4-methyl-cyclohexanol [MCH], 3-octanol [OCT], isopentyl acetate, or ethyl acetate) at constant flow rates of 0.25–0.5 l/min toward the fly’s head.^67^ With the exception of the experiments shown in Figure 4, the four odors were used randomly and interchangeably. Steady-state odor concentrations were estimated with the help of a ppbRAE 3000 photoionization detector (RAE systems); the kinetics of odor concentration changes at the position of the fly were monitored with a 200B miniPID (Aurora Scientific) at 10 kHz. Pressure changes caused by the opening and closing of valves were balanced by a set of pressure-compensating valves and monitored periodically with a mass flow sensor (FBAL001DU, Sensor Technics).

Electric shocks were applied in constant current or constant voltage regimes. In the former, 10-ms current pulses (20–100 μA, Digitimer DS3) were passed through a pair of 50-μm platinum wires touching the fly’s abdomen; in the latter, a printed circuit board (PCB) supporting the fly’s legs was intermittently connected to a 90-V source (Digitimer DS2A). A CCD camera (Guppy F-033; Allied Vision Technologies) equipped with a 3.3x Macro Zoom Lens (Edmund Optics) was used to observe the fly’s reactions and titrate the intensity of the applied current or verify contact with the PCB. PPL1 odor response plasticity was quantified after two training cycles in the constant current regime. Each cycle consisted of a block of twelve 3 s odor pulses at 20 s intervals: six OCT pulses (CS^+^) paired with a single 10-ms current activation at 2 s after TTL-triggered odor onset, and six MCH pulses (CS^–^) without electric shock. Plastic changes in the on and off responses of MBON-γ1pedc>αβ were analyzed after conditioning flies with six 15 s odor pulses, separated by 15 s epochs of clean air, in the constant voltage regime. The PCB was charged twice for 1.5 s, starting at 5.5 and 13.5 s after the TTL-triggered opening of the odor valve (Figure 5C), or at 6.5 and 14.5 s after its TTL-triggered closing (Figure 5D). For optogenetic reinforcement, DAN activity was photostimulated once for 1.5 s, starting at 5.5 s (Figures 6A–6C) or 13.5 s (Figures 6D–6F) after the TTL-triggered odor onset, or at 1.5 s after odor offset (Figures 6G–6I). Measured intensity changes at the antennae lagged by 0.5–2 s behind valve switching commands, with shorter delays at odor offset.

Spikes were detected as maxima in the time derivative of the membrane potential trace; firing rates were calculated as 400-ms moving averages and sampled at 1 kHz. Odor on and off responses were quantified as baseline-subtracted spike rate averages; the mean spike rate in a 1 s window preceding the valve opening command served as the baseline. The measurement windows were anchored to the firing rate peak within 3 s after valve switching. The window for quantifying the on response extended from 250 ms before until 500 ms after the peak; the more transient off response was quantified as the firing rate peak. In spike latency comparisons (Figure 3), occasional trials without spikes at the highest difficulty level (concentration ratio 0.9) were excluded from the analysis.

#### Behavior

The odor choices of male Canton-S flies aged 7–8 days were analyzed individually in transparent plexiglass chambers (50 mm long, 5 mm wide, 1.3 mm high).^9^^,^^23^ Independently controlled (CMOSens, Sensirion) streams of odorless or MCH-infused air entered the chambers at flow rates of 0.25 l/min through ports at the distal ends, converged at the center, and left through lateral vents. PCBs, connected via solid-state relays (Fairchild HSR312L) to a 70-V power supply, served as floors and ceilings. For electric shock reinforcement, the relays were activated once for 2 s, either at 18 s after odor onset, during twelve consecutive 20 s presentations of 2 ppm MCH, which were interleaved with 20 s exposures to air (reinforcing the odor on response; Figure 5A), or once for 2 s, at 18 s after odor offset, during twelve 20 s presentations of air, which were interleaved with 20 s exposures to 2 ppm MCH (reinforcing the odor off response; Figure 5B). Twenty chambers were operated simultaneously in an incubator (Sanyo MIR-154) at 25°C. The chambers were backlit by 940-nm LEDs (TSAL6100, Vishay) and imaged using a Stingray F080B CCD camera (Allied Vision Technologies) with an 18-mm lens (Edmund Optics). A virtual instrument written in LabVIEW controlled the delivery of odors and electric shock and recorded the positions of the 20 flies as functions of time.^23^

Data were processed offline in MATLAB (The MathWorks). Preferences were quantified as the percentages of decisions in favor of MCH or air during 2-min test intervals before and after electric shock reinforcement. Flies making fewer than 2 decisions per test interval were excluded from the analysis.

#### Imaging

Brains of male flies were dissected 2 days after eclosion, fixed in 4% (w/v) paraformaldehyde in phosphate-buffered saline (PBS; 137 mM NaCl, 3 mM KCl, 8 mM Na_2_HPO_4_, 1.5 mM KH_2_PO_4_, pH 7.3) for 20 min at room temperature, washed four times for 15 min in PBS containing 0.2% (v/v) Triton X-100, and mounted in Vectashield (Vector Labs) for imaging native fluoresecence. For immunostaining, fixed brains were permeabilized and blocked in PBS containing 0.2% (v/v) Triton X-100 and 5% (v/v) goat serum for 1 h. To label synaptic structures, the samples were first incubated with mouse monoclonal anti-discs large antibody 4F3 (Developmental Studies Hybridoma Bank, University of Iowa, 1:50) and then with Alexa Fluor 568-conjugated goat anti-mouse IgG (1:200) plus Alexa Fluor 633-conjugated streptavidin (1:150). Each incubation lasted for 48 h at 4°C and was followed by four 20-min washes in PBS. Stained samples were mounted in Vectashield and imaged on a Leica TCS SP5 confocal microscope equipped with an HCX PL APO 40 × /1.3 CS oil immersion objective (Leica). Images were processed in ImageJ.

### Quantification and statistical analysis

Data were analyzed in Prism 9 (GraphPad). Group means were compared by paired, unpaired, or one-sample two-sided t tests, as indicated in figure legends, or by two-way repeated-measures ANOVA, followed by post hoc analyses using Holm–Šídák’s multiple comparisons test. Where the assumption of normality was violated (as indicated by Shapiro–Wilk test), group means were compared using two-sided Wilcoxon or two-sided Mann-Whitney test. Bootstrapped sample distributions and 95% confidence intervals were computed in Python 3.7 using the dabest 0.3.1 software package.^68^

## Data Availability

The published article contains all data generated during this study. Custom instrument control and analysis code used in this study is available from the lead contact.

## Supplemental information

Document S1. Figures S1–S6

Document S2. Article plus supplemental information
