# Supplementary material for: Response competition between neurons and antineurons in the mushroom body
Source: Curr Biol. 2021 Nov 22;31(22):4911–4922.e4. doi: 10.1016/j.cub.2021.09.008 (PMC8612741; doi:10.1016/j.cub.2021.09.008)
Supplement: Document S1. Figures S1–S6 [file mmc1.pdf]

Current Biology, Volume *31*

Supplemental Information

Response competition between neurons and antineurons in the mushroom body

Eleftheria Vrontou, Lukas N. Groschner, Susanne Szydlowski, Ruth Brain, Alina Krebbers, and Gero Miesenböck

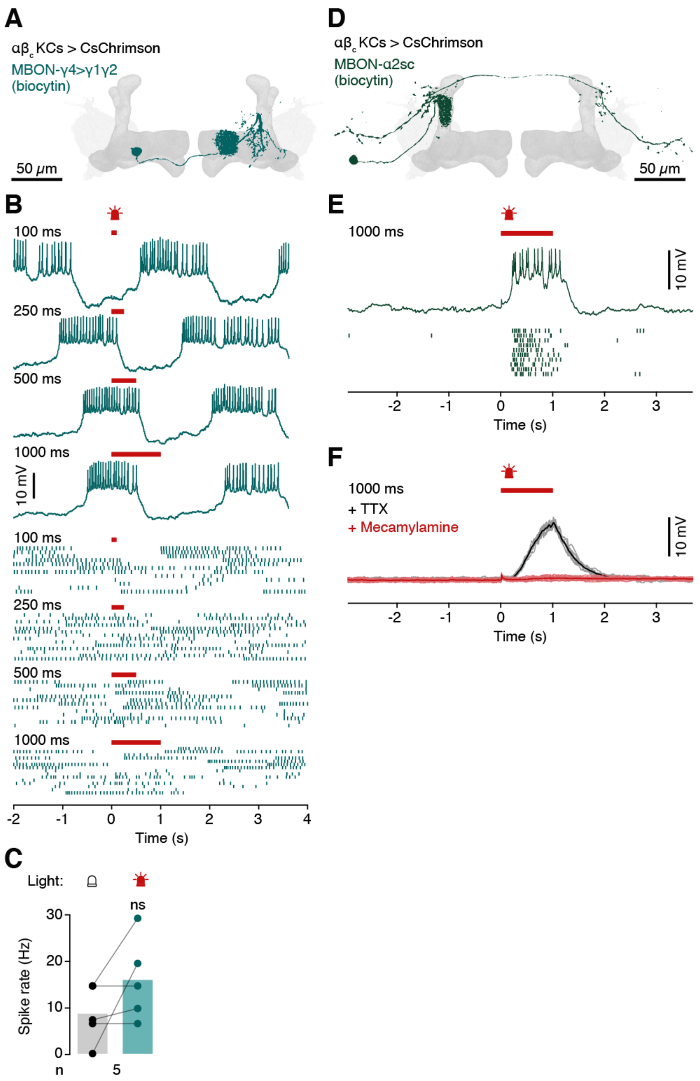

Figure S1. Three types of core-innervating MBON, related to Figure 1

(A) Biocytin fill of MBON-γ4>γ1γ2.

(B) Example voltage traces (top) and spike rasters (bottom) of MBON-γ4>γ1γ2 during optogenetic activation of $\alpha\beta_c$ KCs for the indicated durations.

(C) Average spike rates before and during optogenetic activation of $\alpha\beta_c$ KCs. Illumination left the spike rate of MBON-γ4>γ1γ2 unchanged ($P = 0.2500$; Wilcoxon test). n, number of cells.

(D) Biocytin fill of MBON-α2sc (reproduced from Figure 1A).

(E) Example voltage trace (top) and spike rasters (bottom) of MBON-α2sc during optogenetic activation of $\alpha\beta_c$ KCs for the indicated duration.

(F) Voltage traces of MBON-α2sc during optogenetic activation of $\alpha\beta_c$ KCs in the presence of TTX (black, average; gray, individual trials), followed by the addition of mecamylamine (dark red, average; light red, individual trials).

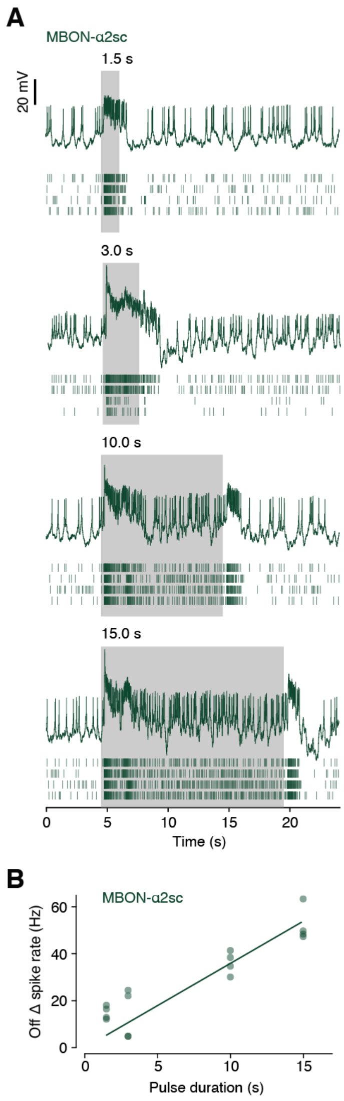

Figure S2. Long odor pulses facilitate the detection of off responses, related to Figure 2

(A) Example voltage traces (top) and spike rasters (bottom) of MBON-α2sc during odor pulses of the indicated durations.

(B) Off responses increased as a function of odor pulse duration (Pearson r = 0.9928, P = 0.0072).

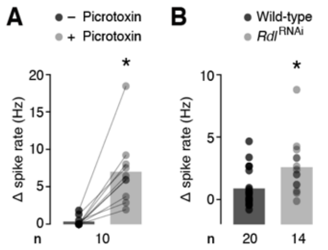

Figure S3. Disinhibition of odor responses in PPL1 neurons, related to Figure 4

(A) Odor-evoked changes in the average spike rates of PPL1 neurons before and after the addition of picrotoxin (P = 0.0020; Wilcoxon test).

(B) Odor-evoked changes in the average spike rates of PPL1-γ1pedc in wild-type flies or flies expressing *MB320C-GAL4*-driven *Rdl* ^{RNAi} (P = 0.0075; Mann-Whitney test). *P < 0.05. Data are means ± SEM; n, number of cells.

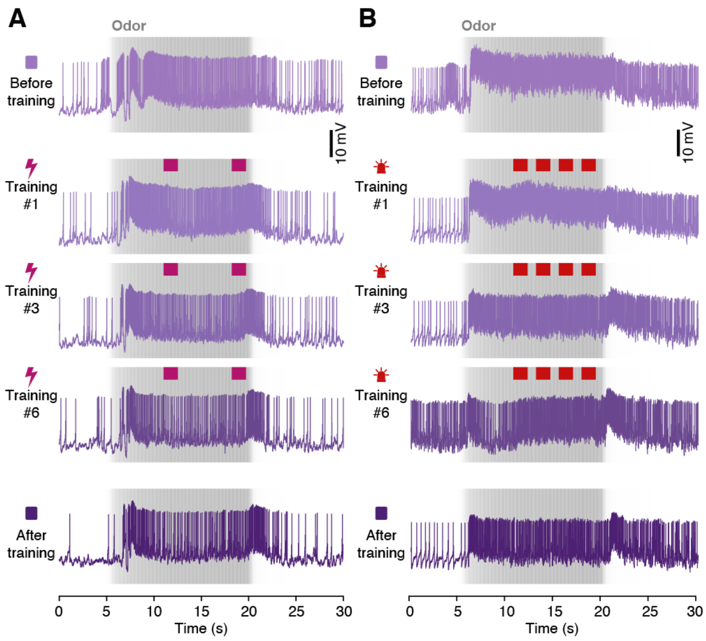

Figure S4. Reciprocal plasticity of on and off responses, related to Figure 5

(A) Example voltage traces of MBON-γ1pedc>αβ during 15-s pulses of odor (gray shading indicates measured concentration time courses) with electric shock reinforcement (magenta bars). The traces depict the evolution of the neuron's on and off responses from the untrained (top) to the trained state (bottom). The same neuron is shown in Figure 5C.

(B) Example voltage traces of MBON-γ1pedc>αβ during 15-s pulses of odor (gray shading indicates measured concentration time courses) with optogenetic activation of PPL1-γ1pedc (crimson bars). The traces depict the evolution of the neuron's on and off responses from the untrained (top) to the trained state (bottom). The same neuron is shown in Figure S5B.

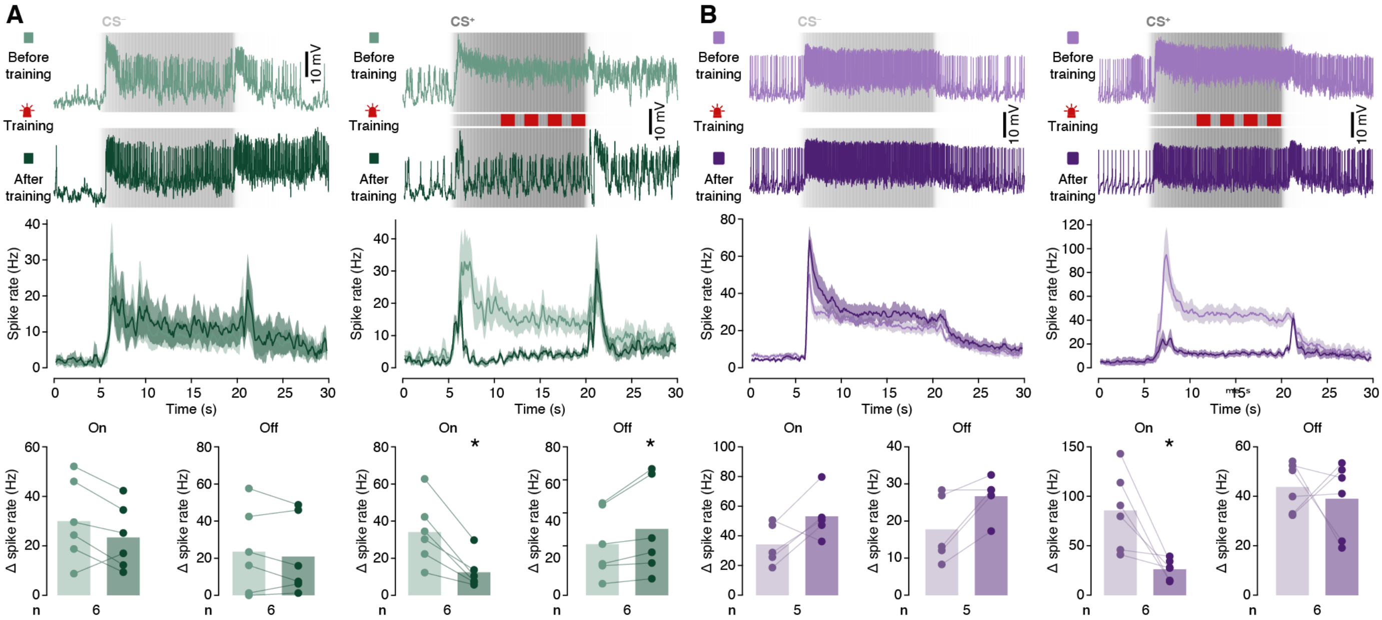

Figure S5. Reciprocal plasticity of on and off responses, related to Figure 5

(A) Responses of MBON-α2sc during 15-s pulses (gray shading indicates measured concentration time courses) of control (CS^-, left) or reinforced odor (CS^+, right), before and after the optogenetic activation of PPL1-α'2α2 (light and dark colors). Top to bottom, example voltage traces, spike rate averages (± SEM), and spike rate changes caused by odor on- and offset. The on and off responses to CS^+ were depressed and potentiated, respectively (P = 0.0312 and P = 0.0312; Wilcoxon tests), while the on and off responses to CS^- were unchanged (P = 0.0938 and P = 0.4375; Wilcoxon tests).

(B) Responses of MBON-γ1pedc>αβ during 15-s pulses (gray shading indicates measured concentration time courses) of control (CS^-, left) or reinforced odor (CS^+, right), before and after the optogenetic activation of PPL1-α'2α2 (light and dark colors). Top to bottom, example voltage traces, spike rate averages (± SEM), and spike rate changes caused by odor on- and offset. The on and off responses to CS^+ were depressed and unchanged, respectively (P = 0.0312 and P = 0.6875; Wilcoxon tests), while the on and off responses to CS^- were unchanged (P = 0.1250 and P = 0.0625; Wilcoxon tests). *$P < 0.05$. Data are means ± SEM; n, number of cells.

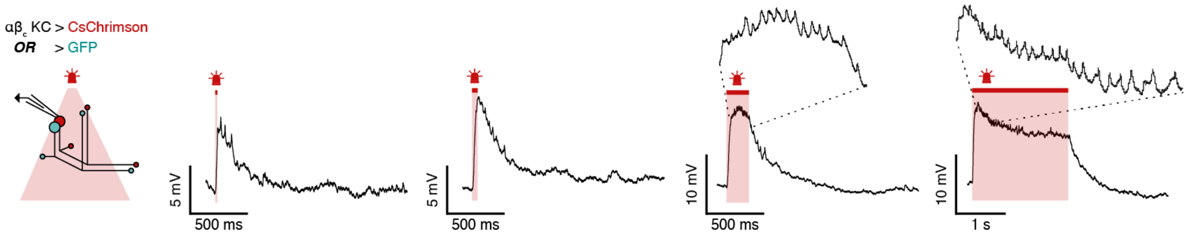

Figure S6. Optogenetic stimulation of αβc KCs, related to Figure 7

Example voltage traces of a CsChrimson-positive αβc KC during light pulses of the indicated durations.
